# Ethnicity coding in a regional cancer registry and in Hospital Episode Statistics

**DOI:** 10.1186/1471-2458-6-281

**Published:** 2006-11-10

**Authors:** Ruth H Jack, Karen M Linklater, David Hofman, Justine Fitzpatrick, Henrik Møller

**Affiliations:** 1 King's College London, Thames Cancer Registry, 1st Floor Capital House, 42 Weston Street, London, UK, SE1 3QD; 2 London Health Observatory, 11-13 Cavendish Square, London, UK, W1G 0AN

## Abstract

**Background:**

The collection of ethnicity information as part of cancer datasets is important for planning services and ensuring equal access, and for epidemiological studies. However, ethnicity has generally not been well recorded in cancer registries in the UK. The aim of this study was to determine the completeness of ethnicity coding in the Thames Cancer Registry (TCR) database and within the Hospital Episode Statistics (HES) data as held by the London Health Observatory, and to investigate factors associated with ethnicity being recorded.

**Methods:**

Records for 111821 hospital admissions of London residents with a malignant cancer as a primary diagnosis between April 2002 and March 2003 and records for 25581 London residents diagnosed with cancer in 2002 were examined. Data on sex, age, cancer network of residence, deprivation, proportion of non-whites in the local authority population, and site of cancer were available. The proportion of patients in each group with a valid ethnicity code was calculated. In the TCR data proportions were also calculated adjusted for all other variables.

**Results:**

Ethnicity was recorded for 90661 (81.1%) of the hospital admissions in the HES data and 5796 (22.7%) patients on the TCR database. Patients resident in areas with a higher proportion of non-white residents and the most deprived populations were more likely to have an ethnic code on the TCR database, though this pattern was not seen in the HES data. Adjustment did not materially affect the association between deprivation and ethnicity being recorded in the TCR data.

**Conclusion:**

There was a large difference in completeness of ethnicity between the data sources. In order to improve the level of recording in TCR data there needs to be better recording of ethnicity in sources TCR data collection staff have access to, or use of information from other sources e.g. electronic data feeds from hospitals or pathology laboratories, or HES data itself supplied directly to TCR. Efforts to collect ethnicity data should be encouraged in all healthcare settings. Future research should explore where the difficulties collecting ethnicity information lie, whether with patients, healthcare professionals or the recording procedure, and how such problems can be overcome.

## Background

Ethnicity is becoming more and more important in understanding the emerging health needs in different communities and to this end the Thames Cancer Registry (TCR) and the London Health Observatory (LHO) are working together looking at ethnicity coding in cancer.

Ethnicity information has been collected in Britain at the last two censuses, 1991 and 2001. In 1991 a simple classification with nine entities was used to show ethnicity: White, Black-Caribbean, Black-African, Black-Other, Indian, Pakistani, Bangladeshi, Chinese, and any other ethnic group. For the 2001 census the classification was changed so that White was expanded to White-British, White-Irish and White-Other, while Asian-Other and four Mixed groups were added to create a total of 16 categories. Cancer registries have included ethnicity as an optional data item since 1993. Ethnicity coding was introduced to the NHS in 1995 as part of the Hospital Episode Statistics (HES) data. Unfortunately the availability of ethnicity data in both datasets has not been improving as rapidly as hoped for, due to a multitude of factors e.g. people are reluctant to collect data they do not feel is being utilised.

Accurate coding and collecting of ethnicity is important for epidemiological research and for planning services. Following the Race Relations (Amendment) Act in 2000, the NHS developed guidance to ensure different ethnic groups have equal access to services. Certain cancers can be associated with certain ethnic groups, for example, breast cancer in Ashkenazi women [1] and prostate cancer in black males [2]. Head and neck cancers are particularly associated with Asians from the Indian sub-continent and nasopharyngeal cancers with the Chinese [3]. Such associations can be due to genetic influences or to lifestyle and environment.

There has not been much research on ethnicity in this country due to the poor availability of ethnicity information. Studies have generally been focused in particular areas and heavily reliant on name algorithms to determine ethnicity [4-7]. Country of birth can also be used as a proxy for ethnicity, but this does not work for older people born in India when it was still part of the British Empire, and it also means that the research is only looking at migrant populations and not the total ethnic group. Migrant populations tend to have an incidence somewhere between the 'home' and the 'host' nation which often approaches the 'host' nation after one or two generations, for example stomach cancers in Japanese people who move to a Western country [2]. Examining these populations is more suitable where immigration is a new phenomenon, and the 'host' population is fairly homogeneous.

The aim of this joint project was to determine the completeness of ethnicity coding on the TCR database and in the HES data as held at the LHO, and investigate factors associated with the availability of ethnicity data.

## Methods

The TCR dataset for the calendar year 2002 and the HES dataset for the financial year April 2002 to March 2003 were examined. Data on whether ethnicity was recorded, sex, age, cancer network of residence, deprivation, proportion of non-whites in the local authority population, and site of cancer were available. The TCR dataset records individual tumours whilst the HES dataset records inpatient episodes. As the datasets covered different time periods and had different definitions of cancer records, no attempt was made to match the datasets to validate the ethnic code information. Ethnicity was regarded as recorded if it was a valid, non-missing code.

The deprivation data was taken from the Index of Multiple Deprivation 2000 income domain [8]. Quintiles were computed for the London area and assigned to records based on postcode of residence.

The proportion of non-whites in the populations of the local authorities in London was calculated from the ONS Labour Force Survey [9] where a quarter of the labour force was surveyed in the summer of 2001 for their ethnicity. This data source was used as Census data were not available at the start of the study. The proportion of the population in each local authority which was non-white was calculated, and the local authorities were grouped into quintiles.

The proportion of patients who had ethnicity recorded was calculated for each variable collected. For the TCR data, logistic regression was then used to fit a fully adjusted model, including sex, age, cancer network of residence, deprivation, proportion of non-whites in the population and site of cancer. Results were then transformed to obtain an adjusted proportion of patients with ethnicity data provided. Tests for trend were done by fitting categorical variables as continuous, and chi^2 ^tests were used to test for heterogeneity.

## Results

Table 1 shows the number and proportion with ethnicity recorded in each dataset. On the HES database, there were 111821 hospital admissions of London residents with a malignant cancer as the primary diagnosis. Ethnicity was recorded for 81.1% (90661) of these admissions; this figure was fairly uniform over sex and deprivation quintile. Ethnicity coding by age-group ranged from 71.8% in the 20–24 year olds to 88.9% in the 5–9 year olds, with the majority of the age groups achieving around 80%. The quintile with the largest proportion of non-whites had the lowest proportion of records with ethnicity coded (76.1%). The highest proportion, 85.3%, was recorded in the middle group. The valid ethnic coding varied between the five networks of residence from 68.2% to 93.0%. Coding over the cancer sites varied from 75.9% in pancreas cancer to 85.2% in bladder cancer.

There were 25581 London residents registered on the TCR database with a malignant cancer (ICD10 C00-C97 excluding basal cell carcinomas of skin) diagnosed in 2002. A total of 22.7% (5796) had a valid ethnicity code (Table 1). The majority of patients with a valid ethnicity had an ethnic code of white, (4652/5796, 80.3%), data not shown. Men were slightly more likely to have a valid ethnic code than women, 23.6% vs. 21.7%. Ethnicity coding varied by age-group between 9.5% in the under 1 year olds to 31.0% in the 5–9 year olds, with the majority of the age-groups achieving around 22%. The patients resident in the most deprived areas were most likely to have an ethnicity code, 33.2% as opposed to 17.0% in the least deprived areas. The availability of ethnicity data ranged from 16.7% to 33.9% with the proportion of non-whites in the population of the local authority (least to most). The availability of ethnicity coding varied between the five cancer networks of residence from 15.6% to 29.9%. Coding over the cancer sites varied from 18.7% in ovarian cancer to 25.7% in head and neck cancers.

The difference in ethnicity coding between the sexes in the TCR data was no longer significant when adjusted for all other variables; this was mostly due to the effect of cancer site as predominantly male cancers (e.g. lung, prostate and bladder) had high proportions of patients with ethnicity recorded (Table 2). As age increased, patients were less likely to have ethnicity recorded, both before and after adjustment. Patients were more likely to have ethnicity recorded as the proportion of non-whites in the population increased. After adjustment this trend was entirely driven by the group with most non-whites having a very high proportion of patients with ethnicity coded, without this group there was a significant negative trend. Adjustment did not affect the associations between cancer networks of residence or deprivation and ethnicity being recorded. In the unadjusted analysis patients with cancer of the head and neck (25.7%) and melanoma of skin (25.6%) had the highest proportion of patients with ethnicity recorded. After adjustment the groups with highest proportion of ethnicity coded were melanoma of skin (27.8%) and bladder cancer (27.5%)

## Discussion

There were large differences between the availability of ethnicity data in the TCR and HES datasets. A number of factors were associated with the likelihood of having ethnicity recorded. Patients resident in areas with a higher proportion of non-white residents and the most deprived population were more likely to have an ethnic code on the TCR database, though this pattern was not seen with the HES data.

Ethnicity information has generally not been well recorded in the UK. However some health data sources have a high level of completeness. The Survey of Prevalent HIV Infections Diagnosed (SOPHID) is a cross-sectional survey of all individuals who have been diagnosed with an HIV infection and attended for HIV related care at an NHS site within a calendar year. Only 5% of these patients seen in London in 2002 did not have ethnicity recorded. [10].

Recording of ethnicity data has been particularly difficult in the TCR database. The proportion of valid ethnic coding on the TCR database for London residents varied by area of residence, deprivation quintile and the proportion of the population that is non-white. The largest variation in ethnic coding in the HES database was between the cancer networks of residence.

The difference in proportions of ethnicity data available in the HES and TCR data may have occurred for a number of reasons. The HES data are downloaded from the hospital patient administration system (PAS) which should include ethnicity. The TCR data come from a number of different data sources, most of which do not have ethnicity as part of the dataset. The primary source of TCR data is a mixture of PAS and pathology data with other data added from radiotherapy or chemotherapy clinic notes. However, some fields, such as ethnicity, may not be available to data collection staff, or staff may not be collecting the data item, viewing it as less important than other variables. This needs to be reviewed internally. Data are also obtained directly from death certificates, GP notes, and outpatient notes. Of all the sources of TCR data, the PAS data is the only source likely to contain ethnicity data which is accessible to the TCR data collection staff.

The variation in ethnicity coding between cancer networks of residence in the HES data is likely to be due to differences in trust of admission. A study of access to revascularisation in London examined the completeness of ethnicity coding for related episodes and found wide variation between hospital trusts. [11].

As the HES dataset records in-patient episodes, rather than individual patients, some patients will be recorded more than once. If these patients were more likely to have their ethnicity recorded, the results would be affected by selection bias, with HES completeness figures artificially inflated. Although the populations being examined are different, this is unlikely to explain the large differences in results found.

In the TCR data the areas with the highest proportion of non-whites in the population were most likely to record ethnicity. Areas with diverse populations may be more aware of the importance of collecting ethnicity information than areas with a large ethnic majority population.

## Conclusion

Improved recording of ethnicity in sources of data that TCR have access to will improve completeness, as will highlighting the importance of collecting ethnicity to data collection staff. Alternatively information from other sources e.g. electronic data feeds or HES data itself supplied directly to the TCR should increase completeness. Efforts to collect ethnicity data should be encouraged in all healthcare settings. Future research should explore where the difficulties collecting ethnicity information lie, whether with patients, healthcare professionals or the recording procedure, and how such problems can be overcome.

## Competing interests

The author(s) declare that they have no competing interests.

## Authors' contributions

RHJ performed the more detailed TCR analysis, interpreted the data and helped to draft the manuscript.

KML was involved in the conception and design of the study, prepared the TCR data, performed the initial TCR analysis and helped to draft the manuscript.

JF was involved in the conception and design of the study, interpreted the data and revised the manuscript.

DH was involved in the conception and design of the study, prepared the HES data, performed the HES analysis and revised the manuscript.

HM was involved in the conception and design of the study, interpreted the data and revised the manuscript.

All authors read and approved the final manuscript.

## Pre-publication history

The pre-publication history for this paper can be accessed here:



## Figures and Tables

**Table 1:** Number and proportion of cancer hospital admissions (HES) and cancer patients (TCR) with ethnicity recorded.

		**Hospital Episode Statistics**			**Thames Cancer Registry**		
		**Coded**		**Total**	**Coded**		**Total**
		**No.**	**%**		**No.**	**%**	
**All cases**		90661	81.1	111821	5796	22.7	25581
**Sex**							
	Male	44887	81.5	55067	3025	23.6	12811
	Female	45774	80.7	56754	2771	21.7	12770
							
**Age groups**							
	<1	161	78.9	204	2	9.5	21
	1–4	1930	87.3	2210	17	24.6	69
	5–9	1532	88.9	1723	13	31.0	42
	10–14	1438	81.6	1762	11	19.0	58
	15–19	1254	78.4	1600	17	23.6	72
	20–24	748	71.8	1042	35	28.0	125
	25–29	1383	78.3	1766	62	24.0	258
	30–34	2100	79.4	2644	99	22.9	432
	35–39	2752	75.5	3647	115	20.5	561
	40–44	3848	80.2	4799	183	24.7	742
	45–49	5122	79.7	6426	234	22.3	1048
	50–54	7683	82.9	9264	330	20.9	1578
	55–59	9568	81.5	11734	520	24.0	2169
	60–64	9930	80.3	12362	604	23.6	2556
	65–69	11217	81.3	13801	724	24.2	2986
	70–74	11078	81.7	13560	852	23.6	3610
	75–79	9199	82.7	11129	815	22.7	3590
	80–84	6037	80.7	7484	633	21.6	2925
	85+	3679	78.9	4660	530	19.4	2739
							
**Ethnicity (London)**							
	Least non-whites	16492	79.9	20642	898	16.7	5392
	2	14520	79.5	18253	760	19.1	3986
	3	18132	85.3	21248	1299	25.3	5133
	4	25026	83.4	29996	1085	18.4	5901
	Most non-whites	16491	76.1	21682	1754	33.9	5169
							
**Network of Residence**							
	North East London	15073	68.2	22094	1318	25.9	5091
	North London	17269	81.8	21103	1247	29.9	4164
	South East London	12895	80.4	16034	887	15.6	5687
	South West London	18200	82.2	22152	887	17.2	5162
	West London	24803	93.0	26661	1457	26.6	5477
							
**Deprivation**							
	Affluent	16304	80.9	20159	973	17.0	5717
	2	18324	81.5	22488	1136	22.9	4958
	3	17795	81.4	21857	1097	21.3	5160
	4	18892	81.5	23181	1049	20.6	5102
	Deprived	19346	80.2	24136	1541	33.2	4644
							
**ICD10 site groups**							
	Head and neck	2448	81.7	2996	232	25.7	904
	Oesophagus	1899	82.6	2298	143	21.6	662
	Stomach	1953	79.4	2461	177	22.7	779
	Colon	7296	84.9	8595	446	24.9	1788
	Rectum	4206	84.5	4978	239	22.5	1060
	Pancreas	1353	75.9	1782	140	19.7	712
	Lung	6587	80.8	8149	853	24.5	3476
	Melanoma of skin	949	79.8	1189	115	25.6	449
	Breast	11937	83.2	14355	759	19.5	3885
	Cervix	1014	78.3	1295	69	21.7	318
	Uterus	702	78.9	890	116	24.4	475
	Ovary	2825	82.4	3430	110	18.7	587
	Prostate	2949	81.1	3637	650	22.9	2843
	Bladder	7253	85.2	8511	215	25.2	854
	Non-Hodgkin's lymphomas	6655	79.5	8371	225	24.0	937
	All other sites	30101	78.8	38203	1307	22.3	5852

**Table 2:** Proportion of cancer patients (TCR) with ethnicity recorded, unadjusted and adjusted for all terms in table.

		**Unadjusted**		**Adjusted**	
		**%**	**(95% CI)**	**%**	**(95% CI)**
**Sex**					
	Male	23.6		23.6	
	Female	21.7	(20.7, 22.7)	23.4	(22.1, 24.7)
**Test for heterogeneity **Chi^2^(1 df)			13.35		0.12
			p = 0.0003		p = 0.7328
					
**Age groups**					
	<1	9.5	(2.4, 31.2)	11.3	(2.8, 35.6)
	1–4	24.6	(15.8, 36.2)	22.9	(14.4, 34.4)
	5–9	31.0	(18.8, 46.4)	31.0	(18.6, 47.0)
	10–14	19.0	(10.8, 31.2)	20.8	(11.8, 34.0)
	15–19	23.6	(15.1, 34.9)	23.8	(15.1, 35.4)
	20–24	28.0	(20.7, 36.7)	29.3	(21.6, 38.5)
	25–29	24.0	(19.1, 29.8)	23.2	(18.2, 29.1)
	30–34	22.9	(19.0, 27.4)	22.3	(18.3, 26.9)
	35–39	20.5	(17.2, 24.3)	20.3	(16.9, 24.2)
	40–44	24.7	(21.4, 28.2)	25.4	(21.9, 29.2)
	45–49	22.3	(19.6, 25.3)	23.4	(20.5, 26.6)
	50–54	20.9	(18.6, 23.4)	21.4	(19.0, 24.0)
	55–59	24.0	(21.8, 26.3)	24.5	(22.2, 26.9)
	60–64	23.6	(21.5, 25.9)	24.0	(21.8, 26.3)
	65–69	24.2	(22.2, 26.4)	24.5	(22.4, 26.7)
	70–74	23.6		23.6	
	75–79	22.7	(20.8, 24.7)	23.0	(21.1, 25.1)
	80–84	21.6	(19.7, 23.7)	22.1	(20.1, 24.3)
	85+	19.4	(17.5, 21.3)	19.8	(17.9, 21.9)
**Test for trend **Chi^2^(1 df)			5.77		5.00
			p = 0.0163		p = 0.0253
					
**Ethnicity (London)**					
	Least non-whites	16.7		16.7	
	2	19.1	(17.5, 20.8)	12.6	(11.3, 14.0)
	3	25.3	(23.5, 27.2)	15.6	(14.1, 17.2)
	4	18.4	(17.0, 19.9)	11.2	(10.1, 12.5)
	Most non-whites	33.9	(31.9, 36.0)	25.5	(23.4, 27.8)
**Test for trend **Chi^2^(1 df)			314.48		76.78
			p < 0.0001		p < 0.0001
					
**Network of Residence**					
	North East London	25.9		25.9	
	North London	29.9	(28.1, 31.9)	42.9	(40.1, 45.7)
	South East London	15.6	(14.4, 16.9)	20.1	(18.5, 21.8)
	South West London	17.2	(15.9, 18.6)	28.2	(25.9, 30.6)
	West London	26.6	(24.9, 28.3)	34.7	(32.4, 37.0)
**Test for heterogeneity **Chi^2^(4 df)			445.92		408.58
			p < 0.0001		p < 0.0001
					
**Deprivation**					
	Affluent	16.5		16.5	
	2	20.4	(18.4, 22.4)	18.3	(16.5, 20.3)
	3	21.7	(19.8, 23.8)	19.0	(17.1, 20.9)
	4	20.8	(19.0, 22.7)	19.6	(17.7, 21.7)
	Deprived	27.6	(25.6, 29.7)	25.3	(23.0, 27.7)
**Test for trend **Chi^2^(1 df)			172.68		91.71
			p < 0.0001		p < 0.0001
					
**ICD10 site groups**					
	Head and neck	25.7		25.7	
	Oesophagus	21.6	(17.8, 25.9)	22.3	(18.3, 26.8)
	Stomach	22.7	(19.0, 26.9)	23.9	(20.0, 28.4)
	Colon	24.9	(21.7, 28.5)	26.2	(22.7, 30.0)
	Rectum	22.5	(19.1, 26.4)	24.1	(20.4, 28.3)
	Pancreas	19.7	(16.2, 23.7)	20.9	(17.1, 25.2)
	Lung	24.5	(21.6, 27.8)	25.3	(22.1, 28.7)
	Melanoma of skin	25.6	(21.0, 30.9)	27.8	(22.8, 33.5)
	Breast	19.5	(17.0, 22.3)	20.9	(18.1, 24.0)
	Cervix	21.7	(17.0, 27.3)	21.3	(16.5, 27.1)
	Uterus	24.4	(20.0, 29.5)	25.5	(20.8, 30.9)
	Ovary	18.7	(15.2, 22.9)	19.4	(15.6, 23.9)
	Prostate	22.9	(20.0, 26.1)	24.0	(20.9, 27.5)
	Bladder	25.2	(21.3, 29.4)	27.5	(23.3, 32.2)
	Non-Hodgkin's lymphomas	24.0	(20.4, 28.1)	25.6	(21.7, 30.0)
	All other sites	22.3	(19.7, 25.3)	23.6	(20.7, 26.7)
**Test for heterogeneity **Chi^2^(15 df)			445.92		408.58
			p < 0.0001		p < 0.0001
